# 3D Hierarchical Porous and N-Doped Carbonized Microspheres Derived from Chitin for Remarkable Adsorption of Congo Red in Aqueous Solution

**DOI:** 10.3390/ijms24010684

**Published:** 2022-12-30

**Authors:** Taimei Cai, Huijie Chen, Lihua Yao, Hailong Peng

**Affiliations:** 1 School of Life Science, Jiangxi Science and Technology Normal University, Nanchang 330013, China; 2 School of Chemistry and Chemical Engineering, Nanchang University, Nanchang 330031, China

**Keywords:** chitin, N-doped carbonized microspheres, congo red, remarkable adsorption

## Abstract

A novel adsorbent of N-doped carbonized microspheres were developed from chitin (N-doped CM-chitin) for adsorption of Congo red (CR). The N-doped CM-chitin showed spherical shape and consisted of carbon nanofibers with 3D hierarchical architecture. There were many micro/nano-pores existing in N-doped CM-chitin with high surface area (455.703 m^2^ g^−1^). The N element was uniformly distributed on the carbon nanofibers and formed with oxidize-N graphitic-N, pyrrolic-N, and pyridinic-N. The N-doped CM-chitin showed excellent adsorption capability for CR and the maximum adsorption amount was approximate 954.47 mg g^−1^. The π-π/n-π interaction, hydrogen-bond interactions, and pore filling adsorption might be the adsorption mechanisms. The adsorption of N-doped CM-chitin was considered as a spontaneous endothermic adsorption process, and which well conformed to the pseudo-second-order kinetic and Langmuir isotherm model. The N-doped CM-chitin exhibited an effective adsorption performance for dynamic CR water with good reusability. Therefore, this work provides new insights into the fabrication of a novel N-doped adsorbent from low-cost and waste biomasses.

## 1. Introduction

Water pollution has become a severe issue in environment due to the rapid expansion in industrialization and urbanization [1]. Among water pollutants, the highly stable toxic dyes in aquatic ecosystems are one of the major pollutants present in the wastewater, which mostly resulted from the leather, textile, food and paint industries [2]. As we all known, dyes contain harmful aromatic structure that make them highly lethal and even carcinogenic to body. Furthermore, dyes are easily broken into benzidine in the aqueous environment and then exhibited high level of toxicity for human body [3,4]. Congo red (CR, Figure S1A,B) is a typical azo anion reactive dye with good solubility in aqueous solution. CR is mainly applied to textiles, printing and dyeing, paper, rubber and plastics industries [5,6]. Nearly 70% of the 10,000 dyes used in textile manufacturing alone are azo dyes [7]. CR displayed severe toxicities of eye stimulator, nausea, vomiting, diarrhea, bold clotting, and drowsiness [6,8]. CR also adversely affect the photosynthetic processes of aquatic creatures because their colored nature reduces the light penetration [9,10]. Therefore, highly efficient removal of CR methods from aquatic ecosystem are necessary.

Generally, CR removal approaches can be divided into chemical, physical, and biological techniques [11,12]. The physical dye sequestration method has been considered as the most efficient method due to the stability of dyes to light, non-biodegradable, and resistant to aerobic digestion treatment [9,13]. Among these physical approaches, adsorption can be regarded as the best technique for CR pollutant removal because of the advantages of low cost, simple operation process, high efficiency, easy recovery, and repeatability [14,15]. Recently, great range of materials can be used as adsorbents for CR removal, such as metal oxides [16,17], Metal–organic frameworks (MOFs) [18], biochars [19], graphenes [20], activated carbons [21], and Zeolitic imidazolate frameworks (ZIFs) [22]. However, these adsorbents generally involved some drawbacks of high-cost and organic solvents during the complicated manufacturing processes, which could lead to unacceptably second pollution for environment and then seriously limit their applications [23,24]. Therefore, fabrication of environmentally friendly, economical, and superior performance adsorbent is necessary. Up to now, lots of renewable biomass resources have been used to development of high-performance adsorbent for pollutant adsorption and removal due to the advantages of low cost, easy of obtainment, nontoxicity to environment, and sustainable development [25]. Thus, functional carbon-based nanomaterials derived from renewable biomass of cellulose, starch, chitosan and chitin are become more and more popular [26,27,28,29].

At present, chitin is mainly produced from discarded shrimp and crab shells during processing of aquatic products. Shrimp shell as shrimp or edible waste is not fully utilized, but the content of chitin in shrimp shell accounts for 15–20%, and the content of chitin in dried shrimp shell is as high as 20–30% [30]. As a natural macromolecular polysaccharide, chitin is received increasing attention because of its properties of biodegradability, biocompatibility, and nontoxicity [31], which widely exists in arthropods (crustaceans, insects, and arachnids), single celled organisms, invertebrates and fish [32,33]. Additionally, chitin contain numerous amino and hydroxyl groups (Figure S1C), and which can be direct converted into nitrogen-doped carbon materials (Figure S1D) via direct carbonization method [34]. Chitin dissolve in NaOH/urea aqueous system at low temperature shows worm-like chains, which can quickly self-aggregate in parallel via hydrogen bonding and hydrophobic interactions to form nanofibers at elevated temperature [32]. The corresponding nanofibrous microspheres were constructed via sol-gel transition method from the chitin nanofibers solution. After carbonization, N-doped CM-chitin formed with hierarchical porosity, a stable 3D interconnected structure, and a high specific surface area [34,35]. Meanwhile, N-doped CM-chitin has the structure of disorder carbon phase with pyridine-N-oxide, graphitic-N, pyrrolic-N, and pyridinic-N [35]. Owing to these advantages, N-doped CM-chitin has been used as adsorbent to removal contaminants from water, such as neonicotinoid residues [35], volatile organic compounds [36], and methyl orange [37]. Up till now, N-doped CM-chitin was just used to statically removal contaminants in stationary vessel. Therefore, N-doped CM-chitin act as the adsorbent for dynamical separation and removal of contaminants might be more meaningful for practical application. Meanwhile, to the best of our knowledge, N-doped CM-chitin was used to adsorption and removal of CR is rarely reported.

Inspired by the previous reports and issues, in this study, an adsorbent of N-doped CM-chitin was fabricated from chitin, which exhibited properties of hierarchical porosity, a stable 3D interconnected structure, and a high specific surface area. The adsorption performance toward CR was explored for the first time, and the kinetic, thermodynamic, and equilibrium were all evaluated for the adsorption mechanism. Meanwhile, the N-doped CM-chitin was also used as adsorbent for CR removal in dynamic water. Therefore, this study may provide new insights into recycling biowaste resources to produce novel adsorbent for contaminants removal from real water samples.

## 2. Results and Discussion

### 2.1. Fabrication of N-Doped CM-Chitin

Chitin, which obtained from sea food wastes, can be easily dissolved in NaOH/urea aqueous solution via a freezing-thawing method [32]. The chitin chains were formed in NaOH/urea solution and can self-aggregate in parallel to form nanofibers via hydrogen bonding and hydrophobic interactions. The corresponding fibrous microspheres derived from chitin (FM-chitin) were developed using a “bottom-up” fabrication method in isooctane phase with surfactants Tween-85 and Span 85 at 0 °C with stirring, and the obtained FM-chitin were induced by heating at 60 °C. The FM-chitin can be directly carbonized to formation of N-doped carbonized microspheres (N-doped CM-chitin), which showed advantages of high surface aera, hierarchical porous structure, different doping N elements, and environmentally friendly processes. Because of these advantages, the N-doped CM-chitin may be a potential adsorbent for pollutants removal in the wastewater treatment field.

### 2.2. Characterizations

**Figure 1:**
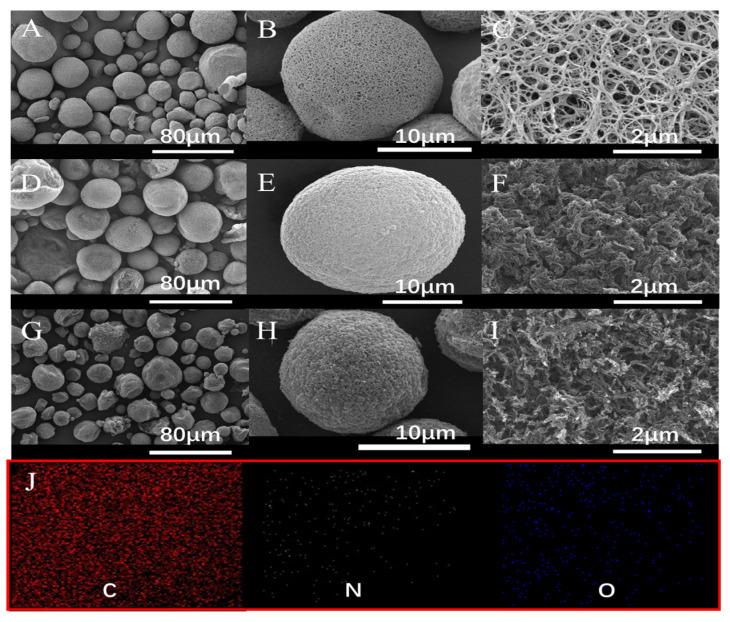
SEM images of FM-chitin (**A**–**C**), N-doped CM-chitin before (**D**–**F**) and after (**G**–**I**) adsorption of CR, and elemental mappings (**J**) of C, N, and O of N-doped CM-chitin.

The morphology of FM-chitin and N-doped CM-chitin was shown in Figure 1, which shows that FM-chitin exhibited a spherical shape with 3D interconnected porous structure and nano/micro-pores on the surface (Figure 1A–C), which may be due to the phase separation induced by the occupying H_2_O during the sol-gel process and the solvent-rich regions contributed to the pore formation [38]. Obviously, there was no essential change in the morphology for N-doped CM-chitin after carbonization (Figure 1D,E), the chitin nanofibers converted into carbon nanofibers with high density after carbonization (Figure 1F), which may be attributed to the shrinkage of nanofibers during the calcining process. Fortunately, the 3D hierarchical porous structure was almost preserved, which was good for adsorption performance. However, the relatively denser surface was observed for N-doped CM-chitin after CR adsorption. The morphology of CR was also investigated (Figure S2), it can be observed that CR showed a good crystal plate morphology. After adsorption (Figure 1G–I), CR crystal cannot be found on the surface of N-doped CM-chitin, suggesting that the native crystalline CR had changed into amorphous CR. It was confirmed that CR has adsorbed onto the fibrous carbon surface of N-doped CM-chitin via the pores and channels. The elements of N-doped CM-chitin were also determined using EDX mapping, showing that the C, O and N elements were uniformly distributed over the carbon nanofibers of N-doped CM-chitin (Figure 1J).

Figure S3A shows the XPS survey of N-doped CM-chitin before and after adsorption of CR, which were carried out to further investigate the elemental contents and bonding states. Three peaks attributed to C1s, N1s and O1s were observed in the full survey spectra of N-doped CM-chitin. A new binding energy peak at 166.6 eV appeared after adsorption of CR, which was owing to the S2p element from CR molecule (Figure S3B). The high-resolution XPS spectra of N-doped CM-chitin before and after adsorption of CR was also measured. The C1s peaks could be deconvolved into the forms of C = O, C-O/C-N, and C-C/C-H at 288.2, 286.3, and 284.8 eV (Figure S3C), respectively. The C-C/C-H became the primary bonding state to form hydrophobic carbon skeleton structure of N-doped CM-chitin. However, C1s exhibited a new peak at the binding energy of 283.50 eV after adsorption of CR (Figure S3D), representing the C = C groups from the phenyl of CR molecule. The O1s spectrum of N-doped CM-chitin before adsorption can be resolved into three peaks at 535.8, 533.3 and 531.7 eV, which was attributed to N-O, O-H, C = O/C-O-C, respectively. After adsorption of CR, a new peak at 531.1 eV of S-O/S = O can be found (Figure S3F). The S-O/S = O bond interaction between surface oxygen functional groups of N-doped CM-chitin and (Φ-N_Azo_ str) groups of CR was beneficial to improve the adsorption capability. The N1 s spectrum can be deconvoluted into the forms of oxidize-N (N^+^O), graphitic-N (N-Q), pyrrolic-N (N-5), and pyridinic-N (N-6), the corresponding peaks was at 403.6, 401.0, 399.1 and 398.2 eV (Figure S3G), respectively. After adsorption, three new peaks at 406.7, 402.9, and 399.9 eV of N = N, C-N, and N-H were appeared (Figure S3H), which may be due to the interaction π–π/n-π and H bonding interactions between N-doped CM-chitin and CR. In conclusion, CR molecules were also adsorbed on the carbon nanofibers surface of N-doped CM-chitin.

**Figure 2:**
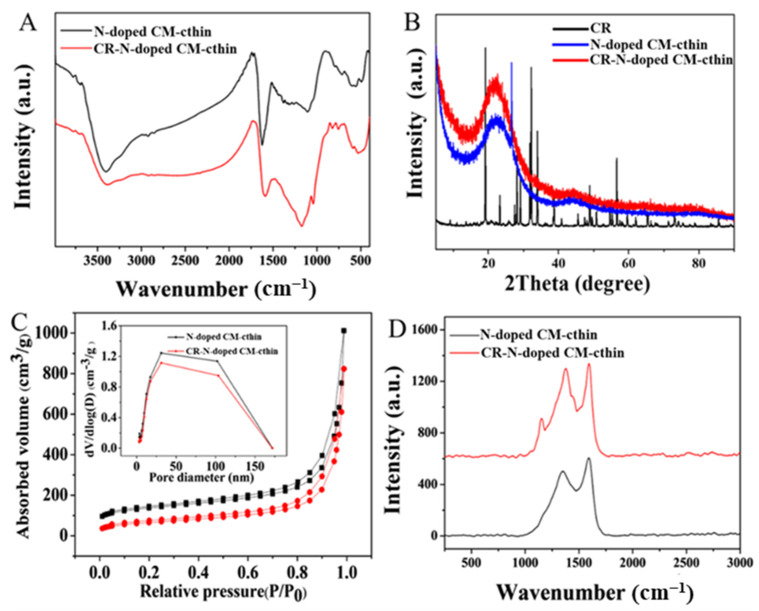
FT-IR spectra (**A**), XRD curves (**B**), Nitrogen adsorption-desorption isotherms (**C**), and Raman spectra (**D**) of N-doped CM-chitin before and after adsorption of CR.

Figure 2A shows the FT-IR spectra of N-doped CM-chitin before and after adsorption of CR. There are some new absorption peaks at 588, 713, 754, and 1172 cm^−1^ also appeared for the N-doped CM-chitin after adsorption of CR. The peak at 588 cm^−1^ was attributed to the shift of out-of-plane flexural stretch vibration peaks of C-H of arene. The peaks of 713 and 754 cm^−1^ were the shift of pure benzylamine bands, and the 1172 cm^−1^ peak was the shift of S-O single bond or S = O double bond, which may be due to the characteristic peaks of sulfo group in CR molecule.

The XRD results of the N-doped CM-chitin and CR were shown in Figure 2B. The N-doped CM-chitin exhibited the similar XRD peaks before and after adsorption of CR. The peaks of 24.30° and 43.21° was corresponded to the (002) and (101) reflection, respectively, which indicated that N-doped CM-chitin have amorphous and graphite crystalline structures [39]. The pure CR exhibited a series of sharp peaks from 10 to 60°, which suggested that CR exhibited the crystalline nature. However, these sharp peaks of CR almost disappeared after adsorption into the N-doped CM-chitin, which indicated that the native crystalline CR had changed into amorphous CR. The XRD results are consistent with SEM, and CR crystals do not appear on the surface of N-doped CM-chitin (Figure S2A,B).

The surface areas of N-doped CM-chitin before and after adsorption were determined using N_2_ adsorption and desorption method (Figure 2C). As can be seen, the N_2_ adsorption and desorption isotherms of N-doped CM-chitin exhibited type Ⅱ: the N_2_ uptakes increase with the increase in relative pressures, especially at high relative pressures, and there is minor hysteresis between the adsorption and desorption isotherms. The specific surface, micropore volume, and total pore volume of N-doped CM-chitin was 455.703 m^2^ g^−1^, 0.098 cm^3^ g^−1^, and 1.565 cm^3^ g^−1^, respectively. However, these porosity parameters of N-doped CM-chitin after adsorption decreased and the value was only 230.919 m^2^ g^−1^, 0.014 cm^3^ g^−1^, and 1.274 cm^3^ g^−1^, respectively. These parameters decreased due to the adsorption of CR molecule on the nanofibrous carbon surface, which is in good agreement with the results of XPS. Meanwhile, the pore size distribution results were determined and shown in Figure 2C (inset), suggesting that N-doped CM-chitin have a meso/macropore dominant hierarchical structure with an extended continuous pore size distribution. This interconnected hierarchical porous nanofibrous architecture would provide more favorable adsorption for CR.

Raman spectra of N-doped CM-chitin before and after adsorption of CR displays in Figure 2D. The peaks at 1357 cm^−1^ and 1591 cm^−1^ were observed for N-doped CM-chitin before adsorption, as well as 1375 cm^−1^ and 1591 cm^−1^ after adsorption, which represent the D and G band of carbon structure, respectively [35]. The D band was attributed to the presence of sp3 carbon atoms, and the G band was related to the in-plane vibration of sp2 carbon atoms [40,41]. The intensity ratio of G band to D band (I_G_:I_D_) presented the graphitization degree of carbon. The I_G_:I_D_ for N-doped CM-chitin was 0.843 and 0.910 before and after adsorption of CR, respectively, indicating that the crystallinity decrease, and the ratio of disorder structures become larger. Meanwhile, a new peak at 1153 cm^−1^ was appeared, which might be due to the azobenzene stretching of CR [42].

### 2.3. Optimization of Adsorption Conditions

**Figure 3:**
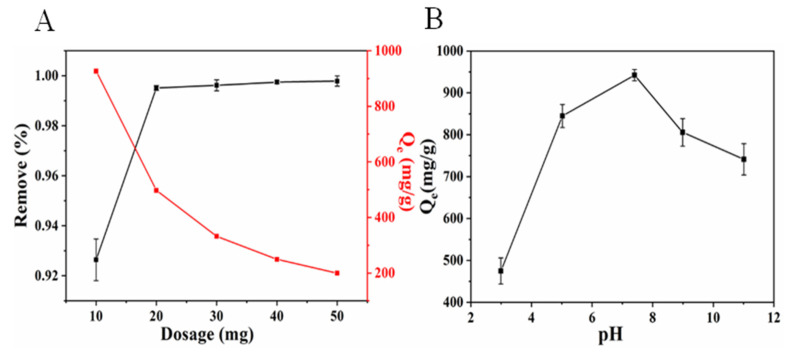
Effect of N-doped CM-chitin dosages on the adsorption capability toward CR (200 mg L^−1^) (**A**), and effect of pH on the N-doped CM-chitin adsorption capability toward CR (200 mg L^−1^) (**B**).

The effect of N-doped CM-chitin amounts (10, 20, 30, 40, and 50 mg) on the adsorption capacity and removal efficiency for CR (200 mg L^−1^, 50 mL) was evaluated (Figure 3A). It can be observed that the removal efficiency increased from 92.64% to 99.79% with increasing the consumption of adsorbent in the same volume of solution. The removal efficiency has less change with adsorbent increasing from 20 mg to 50 mg, and the results also showed that the equilibrium absorption capacity (926.39 mg g^−1^) occurred in adsorbent dose of 10 mg (Figure 3A). The equilibrium absorption capacity decreased from 926.39 mg g^−1^ to 199.57 mg g^−1^ with amount of N-doped CM-chitin increasing from 10 mg to 50 mg, which may be the adsorbents aggregation and overlapping of adsorption sites during the adsorption process [43]. Therefore, 10 mg of the adsorbent dose was used in the following experiments.

The pH value of solution is one of the vital factors in the adsorption capacity due to affecting the surface charge properties of CR and N-doped CM-chitin, and then the electrostatic interaction can be affected under different pH solutions. The CR solution was initially configured with pH of 7.4 and which was set up using 0.1 mol L^−1^ HCl and 0.1 mol L^−1^ NaOH solution. The adsorption capability of N-doped CM-chitin for CR were investigated in different pH solutions with ranging from 3.0 to 11.0. As shown in Figure 3B, the adsorption capacity of CR increased with the pH increasing from 3.0 to 7.4, and the adsorption capability was 474.85 and 942.24 mg g^−1^ at pH value of 3.0 and 7.4, respectively. However, the adsorption capability decreased from 942.24 to 741.31 mg g^−1^ with pH value increasing from 7.4 to 11.0. CR molecule existing in dissociated form as anionic dye ions with negatively charged (dye-SO_3_^−^). Under acidic solution, N-doped CM-chitin protonated to form positively charge sites [44], which resulting in the electrostatic interaction between CR and N-doped CM-chitin. However, the adsorption capability of N-doped CM-chitin not only depended on electrostatic interaction, but also other interactions of hydrogen bonding and π-π interactions. In this study, the N-doped CM-chitin exhibited highest adsorption capability at pH 7.4. Therefore, the pH 7.4 considered as the optimum pH value during the N-doped CM-chitin adsorption processes.

**Figure 4:**
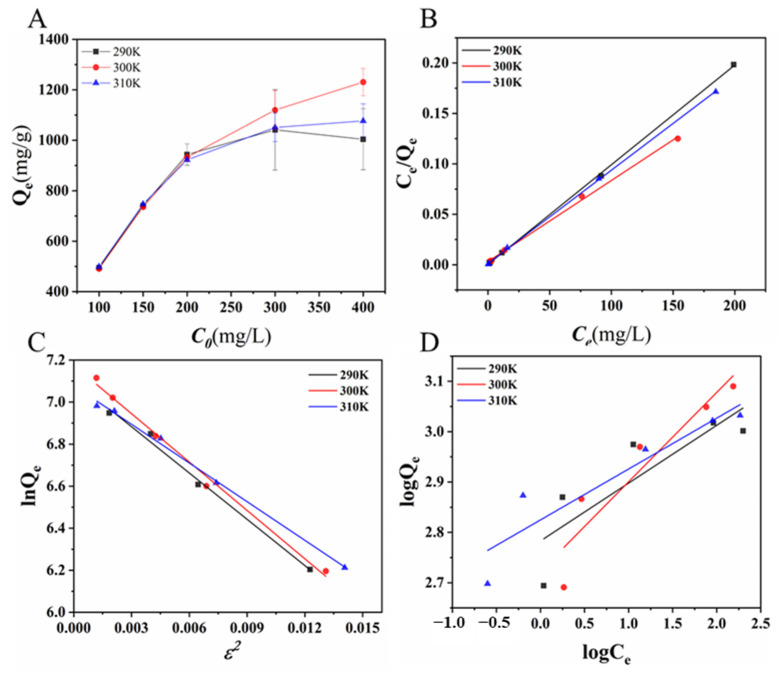
Effect of temperature on the N-doped CM-chitin adsorption capacity (**A**) and adsorption isotherms of CR on N-doped CM-chitin through Langmuir (**B**), Dubinin–Radushkevich (**C**), and Freundlich models (**D**).

The effect of the initial CR concentration (100, 150, 200, 300, and 400 mg L^−1^) on the adsorption capability was also evaluated at pH 7.4 under different temperatures (290 K, 300 K, and 310 K). As shown in Figure 4A, the adsorption capability of CR on N-doped CM-chitin increased from 494.56 to 1003.94 mg g^−1^ (290 K), 490.80 to 1230.74 mg g^1^ (300 K), and 498.75 to 1076.89 mg g^−1^ (310 K) with the concentration of CR increasing from 100 to 400 mg L^−1^. This might be due to two reasons: the increased in initial concentration caused the increase in driving force of the concentration gradient between the bulk solution and N-doped CM-chitin, which could enhance the diffusion of CR into N-doped CM-chitin. Additionally, more collision times occurred between CR and N-doped CM-chitin when increasing CR concentration [45]. In the process of increasing CR concentration, the adsorption capacity of the process increased, the change of adsorption capacity is small and then 200 mg L^−1^ was adopted in subsequent experiments.

### 2.4. Adsorption Isotherms, Kinetics, and Thermodynamics

Figure 4A shows the isothermal adsorption results of CR by N-doped CM-chitin at different temperatures of 290, 300, and 310 K in pH 7.4 solution. To further investigate the adsorption process, three models of Langmuir (Equations (S1) and (S2)), Dubinin-Radushkevieh (D-R) (Equations (S3)–(S5)), and Feundlich (Equation (S6)) were used to fit the isothermal data. The fitting results were described in Figure 4B–D and listed in Table S1. The Langmuir model was better than D-R and Freundlich isotherm models to describe the adsorption of CR into N-doped CM-chitin due to the highest *R*^2^ values at all different temperatures. Meanwhile, the *R*_L_ value was all lower than 1 at different temperatures. The Freundlich constant *n* were larger than 1 and the *E* was lower than 8 kJ mol^−1^. Obviously, N-doped CM-chitin exhibited a monolayer and favorable physical adsorption process.

**Figure 5:**
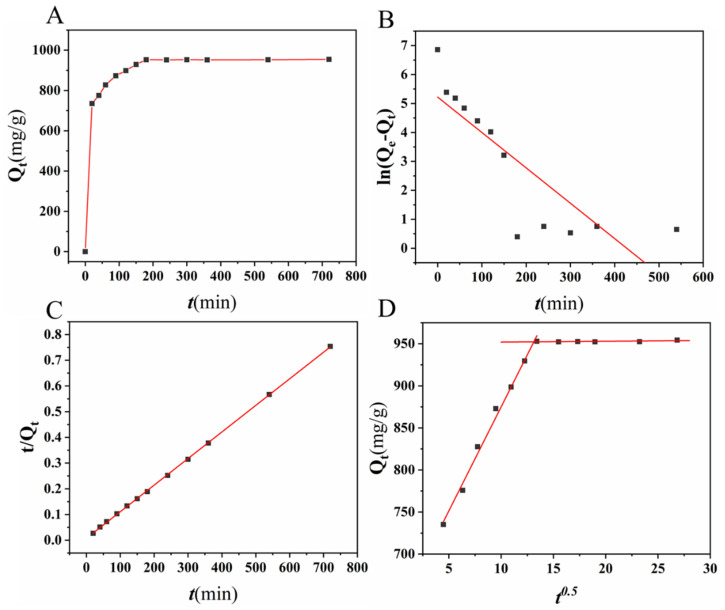
Adsorption kinetics of N-doped CM-chitin toward CR (**A**), Pseudo-first-order kinetic model (**B**), Pseudo-second-order kinetic model (**C**), and Weber-Morris kinetic model (**D**).

Figure 5A shows the kinetic curve of CR adsorption by N-doped CM-chitin at different time. The adsorption capability increased quickly within 90 min, which was due to the hierarchical porous, high surface area, and N-doping structure of N-doped CM-chitin. The adsorption capability attenuated gradually from 90 min to 180 min, and which has no obvious increased after 180 min. After 90 min, the diffusion resistance of CR increased with an increasing amount of CR on the microspheres surface, resulting in a lower adsorption rate. Additionally, the remained CR concentration gradually decreased and the adsorption driving force also gradually decreased, which further decreased the adsorption rate of N-doped CM-chitin for CR after 90 min. Therefore, the kinetic curve of CR adsorption by N-doped CM-chitin was fitted using the pseudo-first order kinetic model (Equations (S7) and (S8) and Figure 5B), pseudo-second order kinetic model (Equations (S9) and (S10) and Figure 5C), and diffusion model (Equation (S11) and Figure 5D) and corresponding parameter results were listed in Table S2. Obviously, Q_e,cal_ (1000 mg g^−1^) obtained from the pseudo-second order kinetic model has good agreement with the experimental result 954.47 mg g^−1^ with the highest *R*^2^ (0.9996), which showed that the adsorption is subject to chemisorption such as hydrogen bond and π-π interactions. The adsorption processes can be expressed by two linear models using the intra-particle diffusion model, showing that the adsorption rate was not only mainly depended on intra-particle diffusion process, but also affected by surface layer adsorption or boundary layer diffusion. As shown in Figure 5D, CR diffuses rapidly on the fibrous carbon surface via the pore channels at the first stage, and instantaneous adsorption occurs on the outer surface of the fibrous carbon of N-doped CM-chitin. The second stage almost horizontal suggesting a dramatic decrease in adsorption rate.

For investigation the effect of temperature on the adsorption of CR on N-doped CM-chitin, the adsorption experiments were carried out at 290, 300, and 310 K. The energy of activations (∆H) and enthalpy change (∆S) was calculated from the Van ‘t Hoff equation (Equation (S12) and Figure S4), the Gibbs free energy change (∆G) was measured from Equation (S13), and the thermodynamic results were listed in Table S3. From the results, ΔH > 0 and negative ΔG, indicating that the adsorption process is endothermic nature and spontaneous. ΔS > 0 showed an entropy increase irregularities at the solid-liquid interface on the carbon nanofibers surface of N-doped CM-chitin.

### 2.5. Effect of Salt Ions

**Figure 6:**
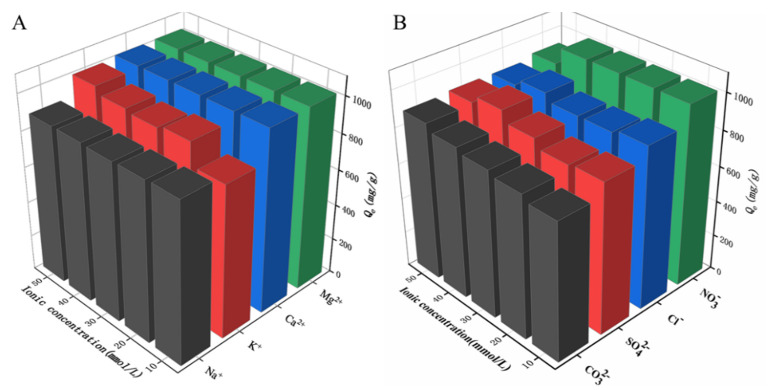
Effect of cations on the adsorption of CR (200 mg L^−1^) onto N-doped CM-chitin (**A**), and effect of coexisting anions on the adsorption of CR (200 mg L^−1^) onto N-doped CM-chitin (**B**).

Previous reports suggest that oxyanions, monovalent and divalent cations present in the waters of an ecosystem could significantly influence the sorption of organic compounds [46]. Figure 6 shows the effect of added cations (Na^+^, K^+^, Mg^2+^ and Ca^2+^) and anions (Cl^−^, NO^3−^, SO_4_^2−^ and CO_3_^2−^) on the adsorption of CR onto N-doped CM-chitin. In the experiment, the ion concentration increased from 10 to 50 mmol L. As shown Figure 6, it was found that cation has a greater promoting effect on N-doped CM-chitin adsorption of CR than anion. For cations, Mg^2+^ ≈ Ca^2+^ > K^+^ > Na^+^ promotes adsorption. The order of the radius of cations is Na^+^ > K^+^ > Ca^2+^ > Mg^2+^, and the larger the radius of cations, the less the polymerization capacity of dyes, and which caused the less promotion of adsorption. For anions, the inhibition of adsorption is CO_3_^2−^ > SO_4_^2−^ > Cl^−^ > NO_3_^−^. With the same valence state, the smaller the hydration radius of ions, the stronger the affinity and the stronger the inhibition of adsorption. Therefore, adsorbent affinity for Cl^−^ is greater than NO_3_^−^.

### 2.6. Possible Adsorption Mechanism

**Figure 7:**
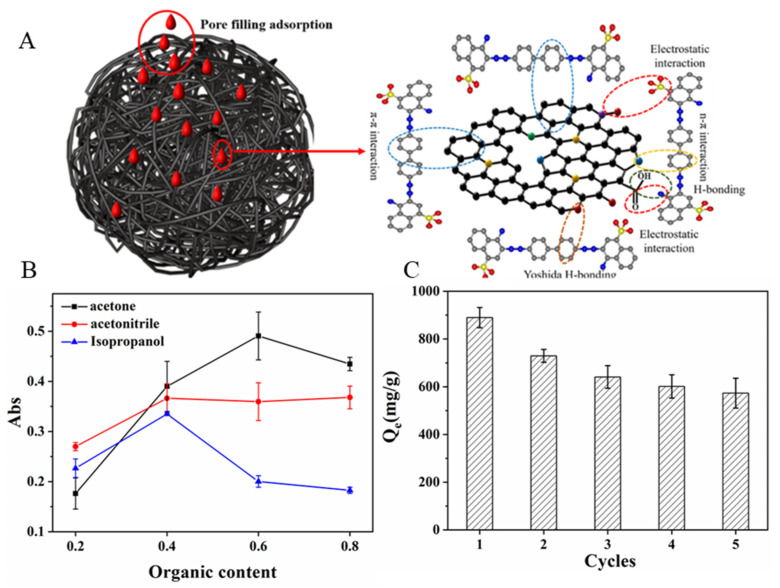
Possible adsorption mechanism of N-doped CM-chitin toward to CR (**A**), desorption rates of different elutant (**B**), and reusability of N-doped CM-chitin (10 mg) for adsorpiton of CR (200 mg L^−1^) at 298.15 K (**C**).

As shown Figure 7A, the possible adsorption mechanism of N-doped CM-chitin was proposed according to the abovementioned characterization results as followings: (1) the π-π and n-π interaction can be formed between the π electrons in N-doped CM-chitin and the benzene/naphthalene ring structure of CR; (2) the hydrogen-bond interactions also can be formed between these carbonyl, hydroxyl and hydroxylamine groups of N-doped CM-chitin and CR; (3) the electrostatic interaction could be happened between the negative charges of CR and positive charges of N-doped CM-chitin; (4) the pore filling adsorption may be also play vital role during the adsorption process [35]. The adsorption capability of N-doped CM-chitin were compared with the other sorbent used in CR adsorption as listed in Table S4, which showed that the adsorption capability of N-doped CM-chitin was better than those of other sorbents in Table S4. The excellent adsorption capability of N-doped CM-chitin was attributed to the multiple adsorption mechanisms, porous structure, and high surface area.

### 2.7. Regeneration and Reuse

The regeneration of N-doped CM-chitin is very important for real application. Thus, five consecutive adsorption-desorption experiments were carried out to evaluate the regeneration performance. In this study, HCl, NaOH, NaCl and acetone solution were used as eluent for N-doped CM-chitin desorption. During the experiments, it was found that the desorption performance of CR by acid, base and salt solution was almost no desorption capability (Figure S5). However, the organic solvent (acetone, acetonitrile, and isopropanol solution) had good effective for the desorption performance (Figure 7B). It can be observed that acetone solution exhibited best elution capability than that of acetonitrile and isopropanol. Therefore, acetone solution (60.00%) was used as the desorption eluent for the N-doped CM-chitin generation. As shown Figure 7C, the adsorption amount exhibited a downward trend with the adsorption times increasing, and the adsorption amount still maintained about 573.15 mg g^−1^ after recycling 5 times. The decreasing adsorption capability might be due to the losing of N-doped CM-chitin during the adsorption-desorption processes, which was consistent with the results of increased disorder of materials shown by Raman spectroscopy. The other reason may be because treatment with acetone solution cannot completely remove CR in the N-doped CM-chitin at the previous adsorption step.

### 2.8. Dynamic Removal of N-Doped CM-Chitin toward CR

**Figure 8:**
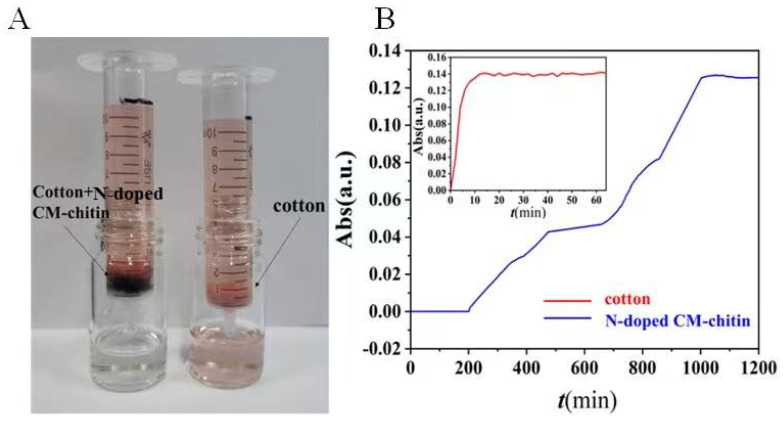
Optical photograph of the dynamic removal of CR (20 mg L^−1^) by cotton + N-doped CM-chitin and cotton (**A**). Breakthrough curves of cotton and N-doped CM-chitin (**B**).

The highly adsorption efficient and the facile application of N-doped CM-chitin are important in the adsorption field. Therefore, dynamic removal experiments were carried out with an N-doped CM-chitin syringe device. As is shown in Figure 8 and Video S1, the real adsorption and removal of CR using N-doped CM-chitin dynamic injector system was evaluated. When CR solution was syringed through the N-doped CM-chitin packing, the CR solution become colorless (Figure 8A). Moreover, the penetration curve of Congo red solution to N-doped CM-chitin is shown in Figure 8B, the N-doped CM-chitin still remain excellent adsorption capability and the CR solution also changed into colorless. Above all, the N-doped CM-chitin exhibited effective adsorption and removal capability for dynamic CR solution, which is suitable for the practical application in water pollution treatment.

### 2.9. Comparison of Adsorption Capacity with Different Sorbents

The adsorption performance of N-doped CM-chitin for CR was compared with other adsorbents and the results were listed in Table S4. It can be seen from Table S4 that the adsorption capacity of N-doped CM-chitin was higher than those of other sorbents. The excellent adsorption capability of N-doped CM-chitin was attributed to the multiple adsorption mechanisms, porous structure, and high surface area. Therefore, N-doped CM-chitin can be applied as a potential adsorbent for practical application in water pollution removal.

## 3. Materials and Methods

### 3.1. Materials

Chitin was supplied by the Golden-shell Biochemical Co., Ltd. (Zhejiang, China) and was purified according to previous report [32,40]. Span 85, isooctane and Tween 85 were purchased from Aladdin (Shanghai, China). CR (>75.00%) was bought from the Solarbio Life Science (Beijing, China). All chemicals used were of analytical grade. Ultrapure water used during the whole processes was manufactured by the Hyperpure water system (Millopore, Bdford, MA, USA).

### 3.2. Fabrication of N-Doped CM-Chitin

Briefly, 94 g of aqueous solution containing NaOH (10.34 g), and urea (3.76 g) was prepared and then stirred continuously for 5 min. After that, 6 g of purified chitin was added into the NaOH/urea solution with stirring for another 10 min, which was placed into a refrigerator at −30 °C for 4 h and then thawed at room temperature. After three cycles of freezing-thawing processes, a transparent and homogeneous chitin aqueous phase was obtained. The oil phase (200 g of isooctane and 8.8 g of Span 85) was added into flask at 0 °C under stirring for 30 min. After that, the chitin aqueous phase was dropped into the oil phase within 5 min and further stirred for 60 min. The emulsification containing 20 g isooctane and 4.8 g Tween85 was added into the flask and stirred for another 60 min. The emulsified chitin solution was transferred to a water bath (60 °C) within 5 min to form the fibers rapidly and woven into microspheres. Subsequently, the fibrous microspheres derived from chitin (FM-chitin) were separated and washed with deionized water and ethanol to completely remove removal of isooctane, Tween-85 and Span-85. The FM-chitin were subjected to solvent-exchange with tertbutanol for 12 h and followed by freeze-dried (−68 °C) for 24 h. Finally, the dried FM-chitin were carbonized under N_2_ atmosphere in a tube furnace heating from room temperature to 650 °C at a rate of 3 °C/min and the equilibrating for 2 h, and then the nitrogen-doped carbonized microspheres derived from chitin (N-doped CM-chitin) were obtained after cooling to room temperature.

### 3.3. Characterizations

The morphology of different samples was observed with scanning electron microscope (SEM) (JSM-6701F, JEOL, Tokyo, Japan). The phase structure of CR before and after adsorption was determined using X-ray diffractometry (XRD, D8 ADVANCE, Bruker, Germany). X-ray photoelectron spectroscopy (XPS) measurements were performed using 2000 a XPS system (ESCALAB250Xi, ThermoFisher Scientific, Waltham, MA, USA). Fourier transform infrared (FT-IR) spectra was recorded on an infrared spectrometer (Nicolet5700, Thermo Nicolet, Waltham, MA, USA). The nitrogen adsorption-desorption isotherms were performed using the Brunauer-Emmett-Teller (BET) (Autosorb-2, Quantachrome, Boynton Beach, FL, USA). Raman spectroscopy was performed using a Confocal Raman microscope (Renishaw, UK).

### 3.4. Adsorption Experiments

Adsorption of N-doped CM-chitin toward CR were performed in the triangle flask was shaken for a rate about 120 rpm under dark condition. The experiment will be repeated three times in parallel to ensure the accuracy of the data. For the effect of the pH value, 10 mg of N-doped CM-chitin was added into 50 mL CR solution with different initial pH (3.0, 5.0, 7.4, 9.0, and 11.0) value for 12 h. The kinetic experiments of N-doped CM-chitin (10 mg) were conducted in CR solution (50 mL, 200 mg L^−1^) and 3 mL solution was withdraw at specific time intervals. For the adsorption isotherms, N-doped CM-chitin (10 mg) was added into CR solutions (50 mL) with concentration ranging from 100 to 400 mg L^−1^. Meanwhile, the dosage effect of N-doped CM-chitin varied from 10 mg to 50 mg for the adsorption capability was also investigated in CR solution (50 mL, 200 mg L^−1^). The CR concentration was measured using UV-vis spectroscopy at 498 nm. The removal efficiency and equilibrium adsorption capacity of CR by N-doped CM-chitin was calculated using the following formulas [16,45]:

(1) $$R\left(\%\right)=\frac{C_{0}-C_{e}}{C_{0}}\times 100\%$$

(2) $$q_{e}=\frac{\left(C_{0}-C_{e}\right)}{M}\times V$$

where R is the removal rate of CR, C_0_ is initial concertation of CR (mg L^−1^), C_e_ is the equilibrium concentration (mg L^−1^), q_e_ (mg L^−1^) is the equilibrium adsorption capacity of N-doped CM-chitin, V is the volume of solution (L), C_e_ is the equilibrium concentration (mg L^−1^), and M is the quantity of N-doped CM-chitin (mg).

### 3.5. Desorption, Regeneration, and Re-Usability of N-Doped CM-Chitin

N-doped CM-chitin (10 mg) was added into CR solution (50 mL, 200 mg L^−1^) and shaken with 120 rpm at 25 °C for 12 h to reach adsorption equilibrium. After filtration, CR on the surface of N-doped CM-chitin was washed using water. After that, N-doped CM-chitin were added into eluents (acetone, isopropanol, and acetonitrile) with desired concentrations and then shaken at 120 rpm at 25 °C for 12 h. The CR concentrations in the regenerated liquid were measured using UV at 498 nm and the desorption efficiency (DE) was then calculated by the following equation [47]:

(3) $$\mathrm{DE}\%=\frac{\left(A_{0}-A_{d}\right)}{A_{0}}\times 100\%$$

where A_0_ is the initial absorbance of CR solution (200 mg L^−1^), A_d_ is the residual absorbance of UV after desorption. For further investigation the regeneration and re-usability, the re-adsorption experiments were carried out in CR solution (50 mL, 200 mg L^−1^) for 5 times abide by the procedure described in adsorption section.

### 3.6. Dynamic Removal of N-Doped CM-Chitin toward CR

Dynamic removal experiments were carried out with syringe device. N-doped CM-chitin were packed into an injector (10 mL), which acted as an adsorption column to form a static bed (50 mg N-doped CM-chitin and 0.2 g cotton). A certain amount of cotton was placed at the bottom of the injector to prevent the outflow of N-doped CM-chitin. The CR solution (10 mL, 20 mg L^−1^) was added into the injector from the top and flowed naturally from the fixed bed. Samples were taken every 3 min. The absorbance was measured with UV-vis spectra and the penetration curve was drawn [48,49].

## 4. Conclusions

In conclusion, a novel adsorbent (N-doped CM-chitin) with 3D porous framework architecture was developed from chitin, and which was used to removal of CR from water for the first time. Different N forms of oxidize-N, graphitic-N, pyrrolic-N, and pyridinic-N distributed in the carbon nanofibers of N-doped CM-chitin. Meanwhile, N-doped CM-chitin had micro/nano-pores and high specific surface area. The N-doped CM-chitin showed excellent removal capability with maximum adsorption amount of 954.47 mg g^−1^ for CR (200 mg L^−1^). The significant adsorption capability might be due to the hydrophobic interaction, electrostatic interaction, π-π/n-π interaction and hydrogen-bond interaction between N-doped CM-chitin and CR. The CR adsorption into the N-doped CM-chitin was considered as a spontaneous endothermic process, and well conformed to the pseudo-second-order kinetic and Langmuir isotherm model. Interestingly, the N-doped CM-chitin also exhibited effective removal capability for the dynamic CR water with long-time stability. Therefore, this study provides new insight into fabrication of novel N-doped adsorbent from low-cost and waste biomass resource, and which has great potential for practical application in water pollution removal.

## Data Availability

Not applicable.

## Supplementary Materials

The supporting information can be downloaded at: https://www.mdpi.com/article/10.3390/ijms24010684/s1. References [50,51,52,53,54,55,56,57,58,59,60,61,62,63,64] are cited in Supplementary Materials.
