# Supplementary material for: 3D Hierarchical Porous and N-Doped Carbonized Microspheres Derived from Chitin for Remarkable Adsorption of Congo Red in Aqueous Solution
Source: Int J Mol Sci. 2022 Dec 30;24(1):684. doi: 10.3390/ijms24010684 (PMC9821205; doi:10.3390/ijms24010684)
Supplement: Supplementary file 1 [file ijms-24-00684-s001.zip › ijms-2075571-supplementary.pdf]

Supplementary

3D hierarchical porous and N-doped carbonized microspheres derived from chitin for remarkable adsorption of Congo red in aqueous solution

Taimei Cai [1], Huijie Chen [2], Lihua Yao [1], Hailong Peng [2*]

[1]*School of Life Science, Jiangxi Science and Technology Normal University, Nanchang 330013, China*

[2]*School of Chemistry and Chemical Engineering, Nanchang University, Nanchang, 330031, China*

*E-mail addressed: penghailong@ncu.edu.cn (H. Peng)

Langmuir, Dubinin-Radushkevieh (D-R), and Freundlich isotherm models

Langmuir model is described using **Eqs. S1,2** [1,2].

$$\frac{C_e}{Q_e} = \frac{1}{k_L Q_m} + \frac{C_e}{Q_m} \qquad 1$$

$$R_L = \frac{1}{1+k_L C_0} \qquad 2$$

where Q_m is the Langmuir maximum adsorption of the CR ($mg{\bullet}g^{-1}$), k_L is the Langmuir constant ($L{\bullet}mg^{-1}$), C_e is the equilibrium concentration of CR ($mg{\bullet}L^{-1}$) and Q_e is the equilibrium adsorption capacity of N-doped NCMs ($mg{\bullet}g^{-1}$). C_0 is the initial CR concentration($mg{\bullet}L^{-1}$). The adsorption behavior is favorable when R_L between 0 and 1, and the adsorption is unfavorable when R_L larger than 1.

Dubinin–Radushkevich (D-R) model is described by **Eqs. S3-5** [1,3].

$$\ln Q_e = lnQ_s - A\varepsilon^2 \qquad 3$$

$$\varepsilon = RT\ln(1 + \frac{1}{C_e}) \qquad 4$$

$$E = \frac{1}{\sqrt{2A}} \qquad 5$$

where Q_e, Q_m and C_e is mentioned in the Langmuir model, A is constant of the D-R model ($mol^2{\bullet}kJ^{-2}$). E is the adsorption energy ($kJ{\bullet}mol^{-1}$). The E was between 8-16 $kJ{\bullet}mol^{-1}$, showing the ion-exchange adsorption, and the physical adsorption occurs when E ranged from 1 to 8 $kJ{\bullet}mol^{-1}$.

Freundlich model is expressed by **Eq. S6** [2].

$$\log Q_e = logk_f + \frac{logC_e}{n} \qquad 6$$

Where k_f is the Freundlich constant related to the adsorption capacity and n is the heterogeneity factor.

Pseudo-first order, Pseudo-second order and Weber-Morris internal diffusion kinetic models.

Pseudo-first order kinetic model is described by **Eqs. S7-8** [4].

$$\frac{dQ_t}{dt} = \mathrm{k}_1(\mathrm{Q}_e - \mathrm{Q}_t) \quad 7$$

$$\ln(Q_e - Q_t) = \ln\ Q_e - k_1 t \quad 8$$

where Q_e is the equilibrium adsorption capacity of N-doped NCMs (mg•g^{-1}); Q_t is the adsorption capacity at time t (mg•g^{-1}); K_1 adsorption rate coefficient of Pseudo-first order kinetic model (min^{-1}); t is the adsorption time (min).

Pseudo-second order kinetic model is described by **Eqs. S9,10** [5].

$$\frac{dQ_t}{dt} = k_2(Q_e - Q_t) \quad 9$$

$$\frac{t}{Q_t} = \frac{1}{k_2 Q_e^2} + \frac{t}{Q_e} \quad 10$$

where Q_e is the equilibrium adsorption capacity of N-doped NCMs (mg•g^{-1}); Q_t is the adsorption capacity at time t (mg•g^{-1}); K_2 adsorption rate coefficient of Pseudo-second order model (g•mg^{-1}•min^{-1});T is the adsorption time (min).

Weber-Morris internal diffusion kinetic models is described by **Eq. S11** [6].

$$Q_t = k_w t^{0.5} + Z \quad 11$$

where Q_t is the adsorption capacity at time t (mg•g^{-1}); K_w is the adsorption rate coefficient of the Weber-Morris internal diffusion kinetic model (mg·g^{-1}·min$^{-0.5}$); t is the adsorption time (min); Z is the adsorption rate constant of the Weber-Morris internal diffusion model.

Adsorption thermodynamics

ΔH (entropy change), ΔS (enthalpy change) and ΔG (Gibbs free energy change) were calculated from following **Eq. S12,13** [1,7].

$$\ln K_\mathrm{d} = \frac{\Delta \mathrm{S}}{R} - \frac{\Delta H}{RT} \quad 12$$

$$\ln \mathrm{K_d} = -\frac{\Delta G}{RT} \quad 13$$

where R is gas constant (8.314J•mol^{-1}•K^{-1}), T is kelvin degree (K), K_d is the equilibrium constant (C_t/C_e). C_t is the concentration of the CR on adsorbent (mg•L^{-1}), and C_e is the equilibrium concentration of CR (mg•L^{-1}).

Figure S1. Structure of CR (A and B), chitin (C), and chitin fibrous carbon (D)

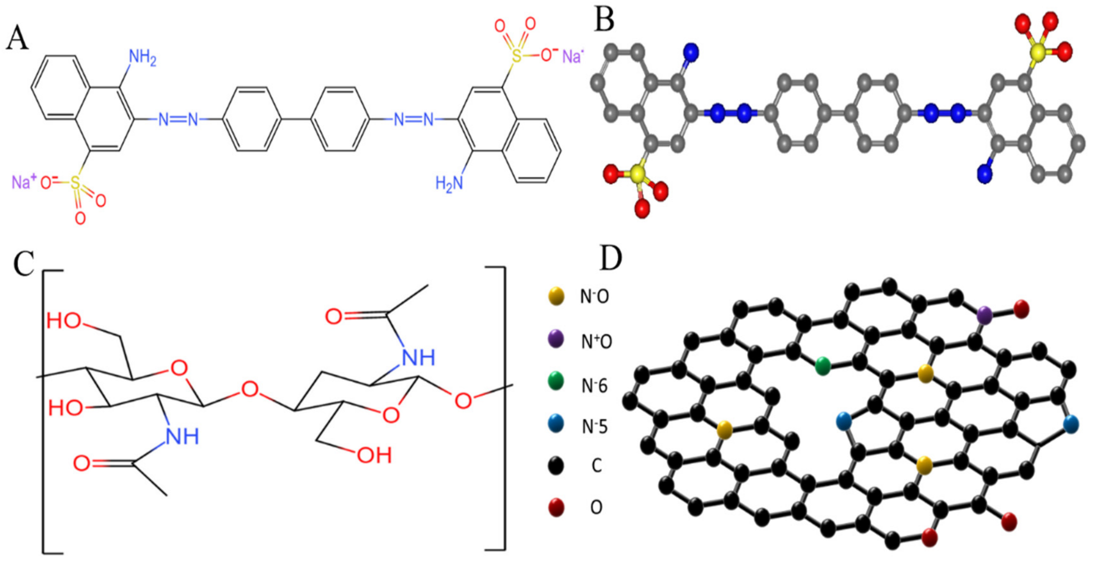

Figure S2. SEM and morphology of CR

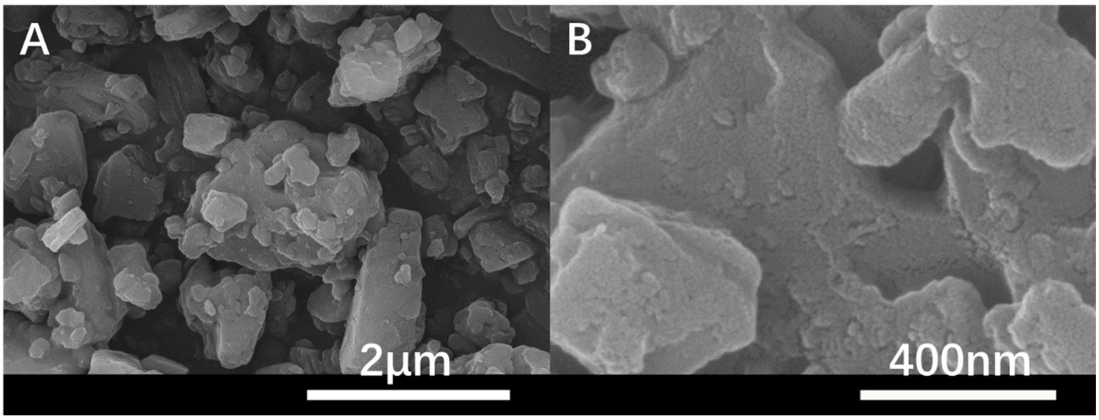

Figure S3. Fully XPS survey spectra of N-doped CM-chitin (A). C1s (C), O1s (E), and N1s (G) split fitting spectra for the N-doped CM-chitin before adsorption. S2p (B), C1s (D), O1s(F), and N1s(H) split fitting spectra for the N-doped CM-chitin after adsorption of CR.

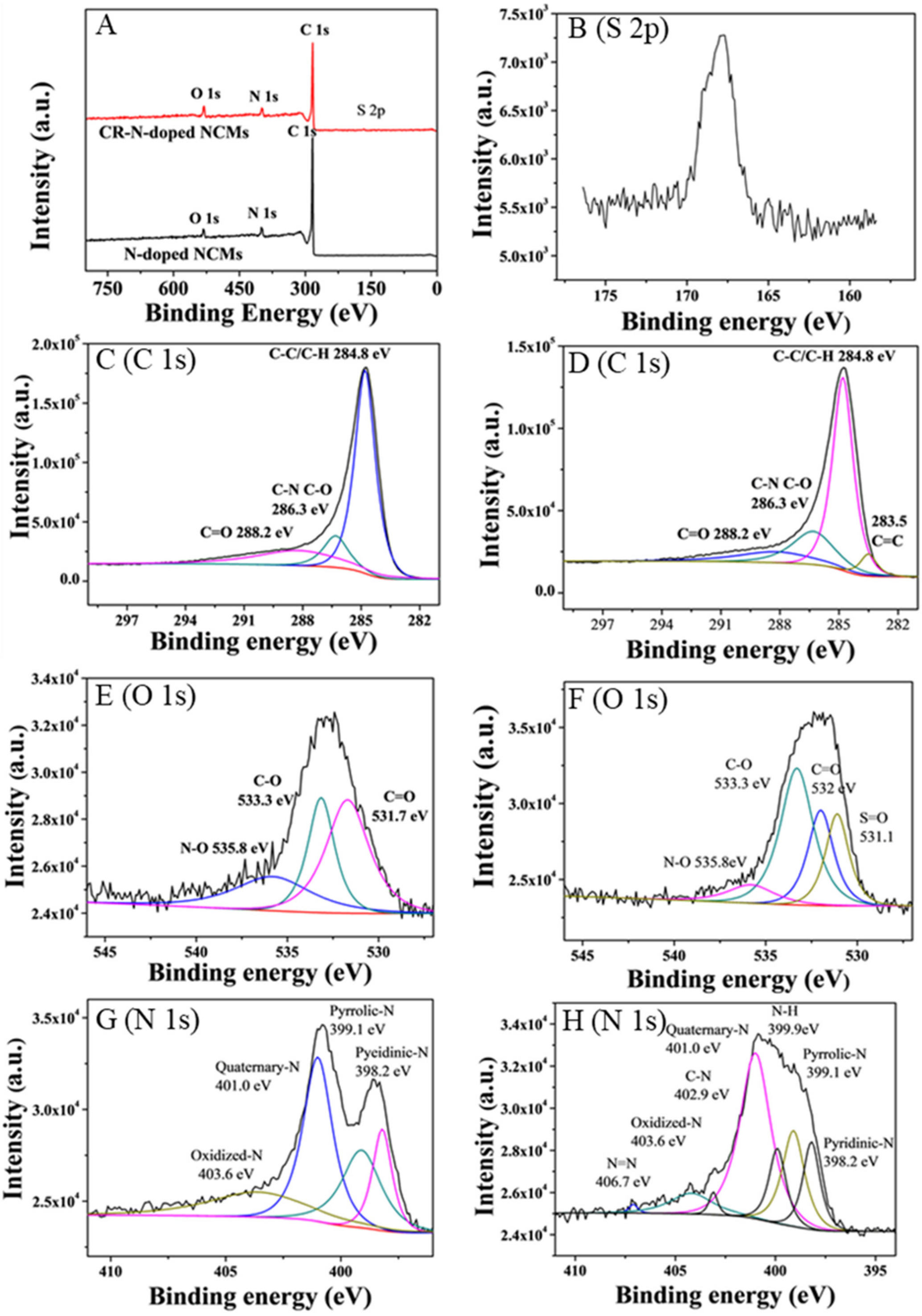

Figure S4. Plot of lnK_d versus 1/T of CR adsorption onto N-doped-NCMs

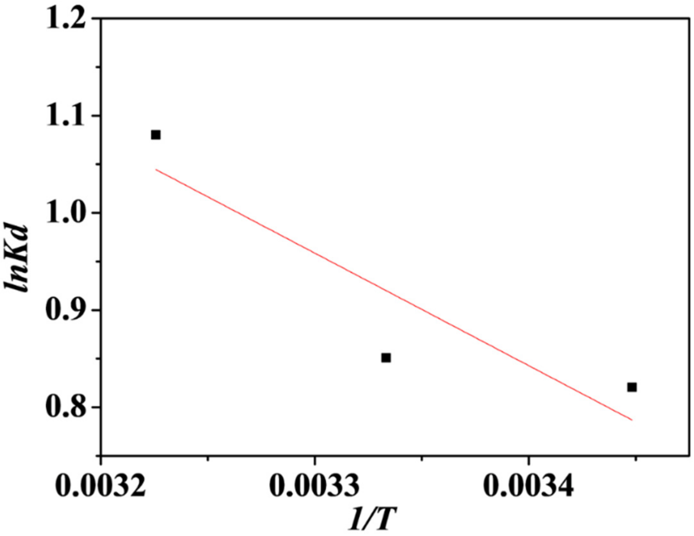

Figure S5. Desorption of N-doped NCMs (10 mg) using different eluents of A (0.1M, HCl) B (0.1M, NaOH), C (0.1M, NaCl), and D (60%, acetone)

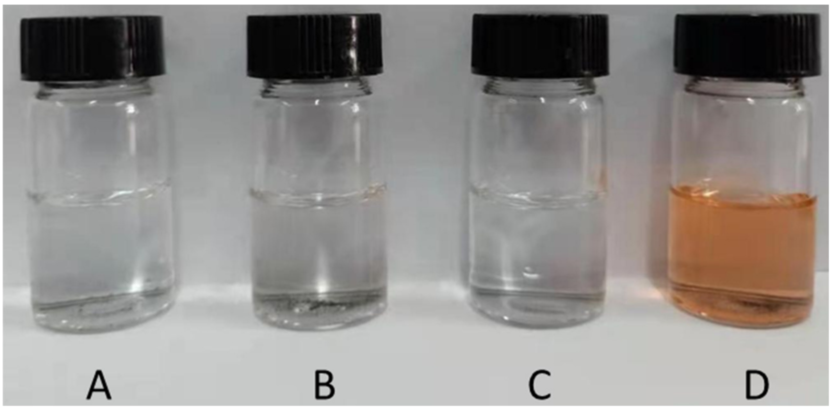

Table S1. Fitting parameters of Langmuir, Dubinin–Radushkevich, and Freundlich isotherm models

Model	Constants	Temperature (K)		
		290	300	310
Langmuir	$Q_{e,exp}$ (mg•g^{-1})	943.33	932.78	922.35
	Q_m (mg•g^{-1})	1012.21	1080.04	1243.71
	R_L	1.6778×10^{-3}	6.2253×10^{-3}	1.8973×10^{-2}
	R^2	0.9996	0.9998	0.9982
Freundlich	K_F	528.4087	667.9439	606.7643
	1/n	0.1774	0.1011	0.1148
	R^2	0.8768	0.8702	0.7352
D-R	Q_s(mg•g^{-1})	1152.75	1306.27	1185.90
	A (mol^2•kJ^{-2})	67.565	76.697	61.328
	E (kJ•mol^{-1})	8.60×10^{-2}	8.07×10^{-2}	9.03×10^{-2}
	R^2	0.9728	0.9930	0.9967

Table S2. Fitting parameters of Pseudo-first-order, Pseudo-second-order and Weber-Morris internal diffusion kinetic models

Experimental	$Q_{e,exp}$(mg•g^{-1})	C_0(mg•L^{-1})	K_1(min^{-1})	$Q_{e,cal}$(mg•g^{-1})	R^2
**Pseudo-first order kinetic model**	954.47	200	0.0122	185.38	0.7050
**Pseudo-second order kinetic model**		C_0(mg•L^{-1})	K_2(g•mg^{-1}•min^{-1})	$Q_{e,cal}$(mg•g^{-1})	R^2
		200	1.05×10^{-4}	1000	0.9996
**Weber-Morris internal diffusion kinetic models**		C_0(mg•L^{-1})	$K_{w,1}$(mg•g^{-1}•min$^{-0.5}$)	Z_1	R_1^2
		200	24.721	627.823	0.9921
		C_0(mg•L^{-1})	$K_{w,2}$(mg•g^{-1}•min$^{-0.5}$)	Z_2	R_2^2
		200	0.154	949.773	0.6222

Table S3. Thermodynamic parameters of N-doped NMCs toward CR adsorption

T(K)	$\ln K_d$	△G($kJ \cdot mol^{-1}$)	△H($kJ \cdot mol^{-1}$)	△S($J \cdot mol^{-1} \cdot K^{-1}$)
290	0.8204	-1.9781	9.6218	39.7226
300	0.8508	-2.1222		
310	1.0802	-2.7840		

Table S4. Comparison of absorption capacity with other sorbents

**Adsorbent**	**q_{max} (mg·g^{-1})**	***Ref***
AlF-rGO	178.57	[8]
Chitosan(CTS)beads	166.67	[9]
sonoenzymolysis of chitin suspension	261.89	[10]
Bentonite/zeolite-NaP composite	46.29	[11]
Chitosan–carbon nanotube(CS/CNT)beads	450.40	[12]
Ca-bentonite	107.41	[13]
SMC(cationic surfactant modified clinoptilolite)	200.00	[14]
cetyltrimethylammonium bromide-acid modified celery residue(CTAB-ACR)	526.32	[15]
hierarchical porous zinc oxide (ZnO)	334.00	[16]
NiO HPHAs	490.20	[17]
tremella-like ferrocene based metal-orgainc framework (TFMOF)	254.14	[18]
ZIF-8$_{DMF}$-M	394	[19]
Ni/Mg/Al layered double hydroxides (NMA-LDHs)	1250	[20]
magnetic polydopamine (PDA)-LDH (MPL)	584.56	[21]
CNF–GnP aerogel	585.3	[22]
**N-doped CM-chitin**	**954.47**	**This work**

References

[1] F. Hou, D. Wang, X. Ma, L. Fan, T. Ding, X. Ye, D. Liu, Enhanced adsorption of Congo red using chitin suspension after sonoenzymolysis, Ultrasonics Sonochemistry. 70 (2021) 105327.

[2] Y. Achour, L. Bahsis, E.-H. Ablouh, H. Yazid, M.R. Laamari, M.E. Haddad, Insight into adsorption mechanism of Congo red dye onto Bombax Buonopozense bark Activated-carbon using Central composite design and DFT studies, Surfaces and Interfaces. 23 (2021) 100977.

[3] J. Zolgharnein, N. Asanjarani, S.N. Mousavi, Optimization and Characterization of Tl(I) Adsorption onto Modified Ulmus carpinifolia Tree Leaves, Clean Soil Air Water. 39 (2011) 250–258.

[4] D. Zhao, Y. Yu, J.P. Chen, Zirconium/polyvinyl alcohol modified flat-sheet polyvinyldene fluoride membrane for decontamination of arsenic: Material design and optimization, study of mechanisms, and application prospects, Chemosphere. 155 (2016) 630–639.

[5] Y.S. Ho, G. McKay, Pseudo-second order model for sorption processes, Process Biochemistry. 34 (1999) 451–465.

[6] S.M. Miraboutalebi, S.K. Nikouzad, M. Peydayesh, N. Allahgholi, L. Vafajoo, G. McKay, Methylene blue adsorption via maize silk powder: Kinetic, equilibrium, thermodynamic studies and residual error analysis, Process Safety and Environmental Protection. 106 (2017) 191–202.

[7] L.B.L. Lim, N. Priyantha, D.T.B. Tennakoon, H.I. Chieng, M.K. Dahri, M. Suklueng, Breadnut peel as a highly effective low-cost biosorbent for methylene blue: Equilibrium, thermodynamic and kinetic studies, Arabian Journal of Chemistry. 10 (2017) S3216–S3228.

[8] Azhdari, R.; Mousavi, S.M.; Hashemi, S.A.; Bahrani, S.; Ramakrishna, S. Decorated graphene with aluminum fumarate metal organic framework as a superior non-toxic agent for efficient removal of Congo Red dye from wastewater. J. Environ. Eng. 2019, 7, 103437.

[9] Raval, N.P.; Shah, P.U.; Ladha, D.G.; Wadhwani, P.M.; Shah, N.K. Comparative study of chitin and chitosan beads for the adsorption of hazardous anionic azo dye Congo Red from wastewater. Desalin. Water Treat. 2016, 57, 9247-9262.

[10] Kammah, M.E.; Elkhatib, E.; Gouveia, S.; Cameselle, C.; Aboukila, E. Cost-effective ecofriendly nanoparticles for rapid and efficient indigo carmine dye removal from wastewater: Adsorption equilibrium, kinetics and mechanism. Environmental Technology & Innovation, 2022, 28, 102595.

[11]Sathishkumar, K.; AlSalhi, M.S.; Sanganyado, E.; Devanesan, S.; Arulprakash, A.; Rajasekar, A. Sequential electrochemical oxidation and bio-treatment of the azo dye congo red and textile effluent. J. Photoch. Photobio. B. 2019, 200, 111655.

[12]Chatterjee, S.; Lee, D.S.; Lee, M.W.; Woo, S.H. Enhanced adsorption of congo red from aqueous solutions by chitosan hydrogel beads impregnated with cetyl trimethyl ammonium bromide. Bioresource Technol. 2009, 100, 2803-2809.

[13]Lian, L.L.; Guo, L.P.; Guo, C.J. Adsorption of Congo red from aqueous solutions onto Ca-bentonite. J. Hazard. Mater. 2009, 161, 126-131.

[14]Nodehi, R.; Shayesteh, H.; Kelishami, A. R. Enhanced adsorption of congo red using cationic surfactant functionalized zeolite particles. Microchem. J. 2020, 153, 104281.
[15]Mohebali, S.; Bastani, D.; Shayesteh, H. Equilibrium, kinetic and thermodynamic studies of a low-cost biosorbent for the removal of Congo red dye: acid and CTAB-acid modified celery (Apium graveolens). J. Mol. Struct. 2019, 1176, 181-193.
[16]Lei, C.S.; Pi, M.; Jiang, C.J.; Cheng, B.; Yu; J.G. Synthesis of hierarchical porous zinc oxide (ZnO) microspheres with highly efficient adsorption of Congo red. J. Colloid. Interf. Sci. 2017, 490, 242-251.
[17]Hu, H.M.; Deng, C.H.; Sun, M.; Zhang, K.H.; Wang, M.; Xu, J.Y.; Le, H.R. Facile template-free synthesis of hierarchically porous NiO hollow architectures with high-efficiency adsorptive removal of Congo red. J. Porous. Mat. 2019, 26, 1743-1753.
[18]Liu, J.Y.; Yu, H.J.; Wang, L. Superior absorption capacity of tremella like ferrocene based metal-organic framework in removal of organic dye from water. J. Hazard. Mater. 2020, 392, 122274.
[19]Liu, J.; Li, J.; Wang, G.; Yang, W.N.; Yang, J.; Liu, Y. Bioinspired zeolitic imidazolate framework (ZIF-8) magnetic micromotors for highly efficient removal of organic pollutants from water. J. Colloid Interface Sci. 2019, 555, 234-244.
[20]Lei, C.S.; Zhu, X.F.; Zhu, B.C.; Jiang, C.J.; Le, Y.; Yu, J.G. Superb adsorption capacity of hierarchical calcined Ni/Mg/Al layered double hydroxides for Congo red and Cr (VI) ions. J. Hazard. Mater. 2017, 321, 801-811.
[21]Li, J.; Fan, Q.H.; Wu, Y.J.; Wang, X.; Chen, C.L.; Tang, Z.Y.; Wang, X.K. Magnetic polydopamine decorated with Mg–Al LDH nanoflakes as a novel bio-based adsorbent for simultaneous removal of potentially toxic metals and anionic dyes. J. Mater. Chem. A 2016, 4, 1737-1746.
[22] Yu, Z.C.; Hu, C.S.; Dichiara, A.B.; Jiang, W.H.; Gu, J. Cellulose Nanofibril/carbon nanomaterial hybrid aerogels for adsorption removal of cationic and anionic organic dyes. Nanomaterials, 2020, 10 (169), 2-20.
